# Medical Journal Association

**Published:** 1881-06

**Authors:** 


MEDICAL JOURNAL ASSOCIATION.—Dr. Langdon B. Edwards, of

Richmond, has been elected President of the Medical Journal Association, and we feel sure he will perform the duties with fidelity and with credit to both himself and the association.

